# Chronic obstructive pulmonary disease phenotype desaturator with hypoxic vascular remodelling and pulmonary hypertension obtained by cluster analysis

**DOI:** 10.1186/2049-6958-7-39

**Published:** 2012-11-05

**Authors:** Domenico Maurizio Toraldo, Mauro Minelli, Francesco De Nuccio, Giuseppe Nicolardi

**Affiliations:** 1 “A. Galateo” Lung Disease Hospital, Rehabilitation Division, Regional Service Puglia, via A. C. Casetti n. 2, San Cesario di Lecce, 73100, ASL, Lecce, Italy; 2 Director of the Operative Unit“IMID Centre” in Campi Salentina Hospital, ASL, Lecce, Italy; 3 Laboratory of Human Anatomy, Department of Biological and Environmental Sciences and Technologies, University of Salento, Lecce, Italy

**Keywords:** Chronic obstructive pulmonary disease, Desaturator, Nocturnal hypoxaemia, Phenotypes, Pulmonary hypertension

## Abstract

Significant heterogeneity of clinical presentation and disease progression exists within chronic obstructive pulmonary disease (COPD). This article discusses and refines the concept of desaturator phenotypes in COPD with pulmonary hypertension (PH) obtained by cluster analysis and presents a pattern of phenotypic markers that could be used as a framework for future diagnosis and research. Nocturnal oxygen desaturation results in sleep disturbances which predispose to nocturnal cardiac dysrhythmias, PH and possibly nocturnal death, particularly during acute exacerbations. We assume that in patients with COPD at least two factors play a role in PH: the severity of pulmonary impairment, and the severity of systemic nocturnal hypoxaemia due to reduced pulmonary functions. Establishing a common language for future research will facilitate our understanding and management of such a disease. This knowledge could lead to different pharmacological treatments and other interventions directed at specific phenotypic groups.

## Background

### Introduction to COPD and its prevalence across the world

Chronic obstructive pulmonary disease (COPD) is characterized by persistent airflow limitation that is usually progressive and associated with an enhanced chronic inflammatory response in the airways and the lungs to noxious particles or gases [1]. The World Health Organization (WHO) has described COPD as a global epidemic, and it is estimated that about 64 million people worldwide are affected by this disease. In 2005 more than 3 million people died because of COPD, accounting for 5% of all deaths worldwide that year. It is estimated that the total number of deaths due to COPD is likely to increase by over 30% in the next 10 years if nothing is done to reduce the risks, particularly those resulting from exposure to tobacco smoke [2]. No effective treatment for COPD exists to date and research into new therapies will be essential if this disease is to be managed in the future.

In recent years, it has emerged that COPD is a complex disease with multiple clinical manifestations and that COPD subjects cannot be described by the severity of airflow limitation alone. Many other independent predictors of clinical outcome have been identified, including worsening dyspnoea, frequency and severity of exacerbations, malnutrition, depression and health-related quality of life (HRQoL) impairment. Therefore, one topic of growing interest in the scientific community, highlighted by studies such as the ECLIPSE [3], is the identification of different phenotypic subgroups of patients. It is possible that the different phenotypes are characterized by different etiopathogenetic mechanisms and that patients in different subgroups will respond differently to drug therapy.

This clinical commentary focuses on COPD and nocturnal hypoxaemia, which can be the cause of pulmonary hypertension (PH). This condition may represent a new phenotype of COPD the desaturator characterized by cluster analysis, a new method of statistical analysis. Cluster analysis is used as artificial intelligence in such diverse fields: biology, medicine, psychology, and business. It entails grouping similar objects into distinct, mutually exclusive subsets referred to as clusters. Elements within a cluster share a high degree of “natural association”, whereas the clusters are relatively distinct from one another. This procedure has recently been used in clinical medicine to classify patients and their data.

We assume that in patients with COPD and PH at least two factors play a role: the severity of pulmonary impairment, and the severity of nocturnal hypoxemia due to reduced pulmonary functions. The severity of COPD also influences the degree of oxygen desaturation and the lower the FEV1/FVC ratio, the more likely is the possibility of significant desaturation during sleep.

### Scientific knowledge on the issue

1) Epidemiological data suggest that nocturnal symptoms and nocturnal oxygen desaturation with symptomatic sleep disturbance are common and may occur in more than 76% of patients with COPD.

2) COPD patients with a T_90_ of ≥ 30%, a mean nocturnal arterial oxygen saturation (SatO_2_) of ≤ 90% and a minimum (nadir) SatO_2_ of 85% have been defined as desaturators (D) and the others as non-desaturators (ND).

3) The worsening of nocturnal hypoxaemia is due to a variable combination of alveolar hypoventilation and ventilation-perfusion mismatching.

4) Nocturnal oxygen desaturation appears to contribute to the development of pulmonary hypertension (PH). PH is defined as an increase in mean pulmonary arterial pressure (MPAP) ≥ 25 mmHg at rest as assessed by right heart catheterization (RCH).

5) Poor correlation between lung function parameters and pulmonary arterial pressure (PAP) suggests that factors other than airway obstruction and loss of alveolar surface area may play a role in PH.

## Main text

### The complexity of COPD/COPD as a systemic disease

The recognition of COPD as a systemic disease has developed in recent years [4]. Comorbidities such as chronic heart failure, cardiovascular disease, depression, diabetes, muscle wasting, weight loss, lung cancer, and osteoporosis can frequently be found in patients with COPD and are considered to be part of the commonly prevalent non-pulmonary sequelae of the disease [5,6]. Systemic inflammation is considered a hallmark of COPD and may be one of the key mechanisms responsible for the increased rate of comorbidities [7].

Even though more than 75% of patients with COPD report poor sleep quality, night-time symptoms and sleep disturbances in COPD are generally not considered in the clinical management of the disease [8]. For instance, the most recent Global Initiative for Chronic Obstructive Lung Disease guidelines (GOLD 2011) do not mention sleep disturbance as a target for therapeutic intervention, and no or very limited specific guidance is offered on appropriate management strategies or pharmacological interventions for patients with COPD who report alterations in sleep. In fact, the nature and cause of disturbed sleep in COPD has been poorly characterized so far, and the long-term clinical consequences of sleep disturbance in COPD have yet to be explored.

In general, COPD-associated PH is mild and develops in patients with a severely impaired pulmonary function. PH may develop slowly in the natural course of COPD. The main cause is hypoxic vasoconstriction of small pulmonary arterioles that subsequently undergo structural changes, reducing their elasticity and cross-sectional surface area [9]. Nocturnal hypoxaemia appears to contribute to the development of PH, even in the absence of significant awake hypoxaemia [10].

### Sleep disturbance and nocturnal oxygen desaturation

Sleep plays a key role in COPD, although the degree of nocturnal oxygen desaturation during sleep varies from patient to patient. Usually, in patients with COPD, there is decreased sleep efficiency, decreased total sleep time, and increased wake time after initial sleep onset. In addition, there is decreased Rapid Eye Movement (REM) sleep and decreased stages III and IV sleep. These changes in sleep are more pronounced in elderly patients with COPD.

**Figure 1:**
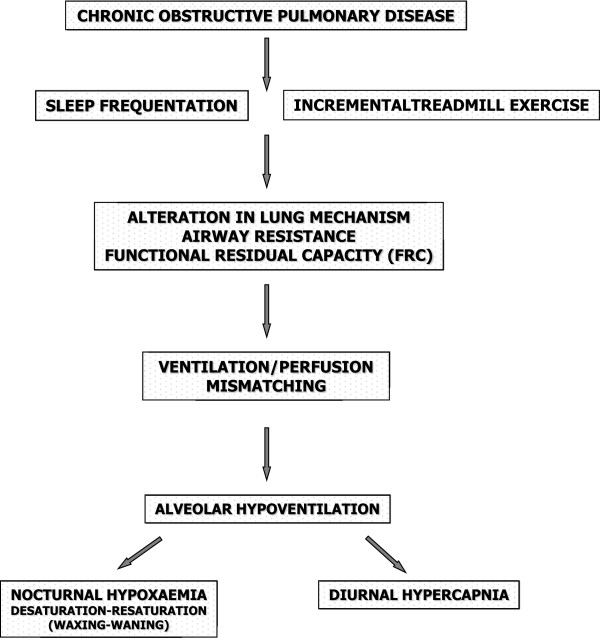
Pathophysiological effects of sleep on respiration in COPD patients with chronic nocturnal hypoxaemia.

The nocturnal oxygen desaturation in COPD patients is determined by the daytime arterial saturation. One study [11] showed that, in COPD patients, breathing difficulties during sleep are the most common symptoms after dyspnoea and fatigue [12]. Another study [13] confirmed a reduction in the amount and quality of sleep in COPD patients compared with healthy controls. Patients with COPD who are hypoxaemic during wakefulness become more hypoxaemic during sleep [13]. The worsening of nocturnal hypoxaemia is due to a variable combination of alveolar hypoventilation and ventilation-perfusion mismatching. The respiratory muscle contribution to breathing is reduced in sleep, which results in a decreased functional residual capacity (FRC) and, with a relative increase in the physiological dead space, leads to hypercapnia and hence acidosis [14]. The consequences of sleep-related hypoxaemia include peaks of PH due to hypoxic pulmonary vasoconstriction, generally observed in patients with marked daytime hypoxaemia [14] (Figure 1). 

Nocturnal hypoventilation has been demonstrated in COPD during REM sleep, with associated oxygen desaturation [15]. A correlation has been shown between the awake arterial oxygen tension (measured as the partial pressure of oxygen in arterial blood (PaO_2_) in mmHg) and nocturnal arterial oxygen desaturation (measured as the percentage of oxygen saturation in arterial blood (SatO_2_), particularly during REM sleep; in addition, the nocturnal desaturation is accompanied by hypercapnia [16,17]. The contribution of respiratory muscle hypotonia to sleep-related breathing is reduced in sleep, particularly during REM. This contributes to a worsening of ventilation-perfusion mismatching, which also aggravates hypoxaemia and hypercapnia. The consequences of nocturnal hypoxaemia (and hypercapnia) in patients with COPD may include acute events, such as altered sleep structure, arrhythmias and increased risk of cardiovascular and cerebrovascular disease [18].

### Hypoxemia and COPD-associated pulmonary hypertension

The exact prevalence of PH in patients with COPD is unclear [19], although it is more common and more severe in patients with advanced COPD who show severe long-term hypoxaemia. Even though PH is mild to moderate in most COPD patients, it can markedly worsen during acute exacerbations, sleep and physical exercise, as shown in a study by Kessler et al. [20] in which 25% of COPD patients developed PH during physical activity. These acute increases in PH can facilitate the development of right heart failure (RHF), so it is important to diagnose PH as soon as possible in high-risk COPD patients. The diagnosis of PH in COPD patients is difficult. PH should be suspected in patients with COPD and declining functional capacity or increasing shortness of breath in the presence of stable airflow obstruction and the lack of an alternative explanation (eg. interstitial lung disease, obstructive sleep apnea, or other comorbidities).

The published studies differ not only in their definition of PH but also in the conditions under which PH is reported (rest, exercise and exacerbation). According to the European Society of Cardiology and the European Respiratory Society, PH is defined as an increase in mean pulmonary arterial pressure (MPAP) ≥ 25 mmHg at rest as assessed by right heart catheterization (RHC) [21]. This is the definition adhered to by the authors of this commentary. Nocturnal oxygen desaturation seems to contribute to the development of PH, even in the absence of significant daytime hypoxemia [22]. REM-associated falls in SatO_2_ are associated with increases in pulmonary arterial pressure (PAP) which can be reversed by supplemental oxygen, although most COPD patients with sustained PH are also hypoxaemic during the daytime. Various arrhythmias are also reported during episodes of nocturnal desaturation [23]. These consequences might help to explain why nocturnal oxygen desaturation is a marker of increased mortality, and why COPD patients are reported to die more frequently at night than expected [24]. COPD patients have increased circulating markers of systemic inflammation when compared with healthy controls [25]. Systemic inflammation stimulates pulmonary vasculature and promotes the development of skeletal muscle dysfunction and osteopenia [26]. The pulmonary vasculature of COPD patients with PH is characterized by luminal narrowing due to thickening of the intima, along with arteriolar muscularization.

Another major contributor to the development of PH in COPD patients is nocturnal hypoxia-induced remodelling of the pulmonary vasculature [27]. Hypoxia-induced pulmonary vasoconstriction is a protective response that keeps the ventilation-perfusion ratio optimal by shunting blood away from hypoxemic areas. The traditional hypoxic model of PH is based on the hypothesis that chronic hypoxia initiates vascular remodelling leading to permanent changes in pulmonary vasculature [28]. Studies performed *in vitro* have elucidated the mechanisms underlying hypoxia-driven vascular changes [29,30]. Barbera et al. [31] evaluated COPD patients undergoing lung resection and demonstrated that vascular changes contribute to vascular remodelling and may have an effect on vascular dynamics leading to PH. Nocturnal hypoxia may also induce endothelial cells to release proliferating cytokines leading to cellular hypertrophy in the vessel wall and an increase in the production of extracellular matrix proteins [32]. Although one recent work [33] has shown that there is no correlation between the severity of airway obstruction hypoxemia and PH, there seems to be a large body of evidence to support the role of hypoxemia-induced remodelling of pulmonary vasculature in the development of PH.

## Discussion

In some studies [34-36], COPD patients have been categorized as desaturators (D) or non-desaturators (ND) based on the levels of oxygen desaturation defined by certain parameters, namely SatO_2_ and T_90_. Nocturnal hypoxemia has been defined as a SatO_2_ of ≤ 90% for at least 5 min with a nadir SatO_2_ of ≤ 85%. The percentage of nocturnal total recording time (TRT) has been defined as “time spent in bed – sleep latency + intra-sleep wakefulness”. TRT with a SatO_2_ of ≤ 90% has been defined as the T_90_ percentage. The minimal TRT required for a satisfactory analysis of nocturnal recordings was 2 hours. COPD patients with a T_90_ of ≥ 30% and a nadir SatO_2_ of 85% have been defined as D patients and the others as ND patients. D patients are more at risk of respiratory complications such as cardiovascular disease, hospitalization, and ultimately death [37].

In this commentary, the authors describe their own studies in which cluster analysis was used to redefine the COPD D phenotype and show that daytime clinical variables can be predictive of the severity of nocturnal oxygen desaturation in D patients [34]. Cluster analysis refers to the use of various algorithms to place individuals into different groups or “clusters”, based on multiple measured parameters, such that individuals in the same cluster are more similar to each other than to those in different clusters. In 29 out of 51 consecutive COPD outpatients [22] with mild daytime hypoxemia (PaO_2_ 60–70 mmHg), cluster analysis identified a pattern of daytime clinical variables that distinguished D patients from ND patients. Considering all patients, the values of T_90_, MPAP at rest, partial pressure of CO_2_ in the blood (PaCO_2_), nadir nocturnal SatO_2_, mean nocturnal SatO_2_ level, predicted total lung capacity (TLC), baseline awake SatO_2_ level, predicted vital capacity (VC), and body mass index (BMI) had a bimodal distribution. The variables examined in the study did not differ between men and women. Rather than using T_90_ alone, the study showed that D patients may be identified by a pattern of T_90_ (30.08%–45.1%, P = 0.0001), MPAP (33.1 ± 0.7 mmHg, P = 0.0006), and PaCO_2_(35.0–57.9 mmHg, p = 0.0005) values, with the latter two variables being predictors of the severity of nocturnal oxygen desaturation.

**Figure 2:**
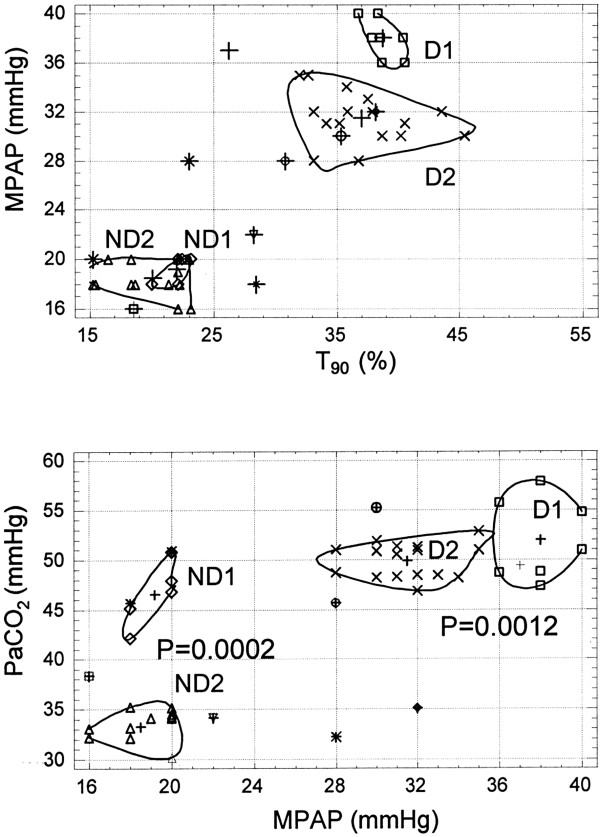
**Correlation obtained by cluster analysis, between mean pulmonary arterial pressures made by echocardiography versus diurnal PaCO**_**2**_**and nocturnal T90 values.** (D) Desaturator patients may be identified, by cluster analysis, by a pattern of Mean PAP and, rather than by T90 alone, with the latter two variables being predictors of nocturnal desaturation severity. From [35].

Cluster analysis has also identified a subgroup of COPD D patients in which parameters describing PH and parameters describing respiratory dysfunction are linked. These data also correlate with BMI, PaCO_2_, VC and TLC. In which case, the parameters describing respiratory dysfunction (PaCO_2_, VC, TLC) and BMI could be used to predict PH. Our recent article [38] defined the COPD D phenotype with PH as a new disease phenotype (Figure 2). 

In contrast, Chaouat and co-workers [39] showed that isolated nocturnal hypoxaemia does not induce permanent PH, nor leads to worsening levels of daytime blood gases. In fact, it is now recognized that the development of PH due to chronic nocturnal hypoxaemia requires a certain threshold of severity and duration. It is likely that this threshold was not reached during the episodes of nocturnal hypoxaemia in D patients this study.

**Figure 3:**
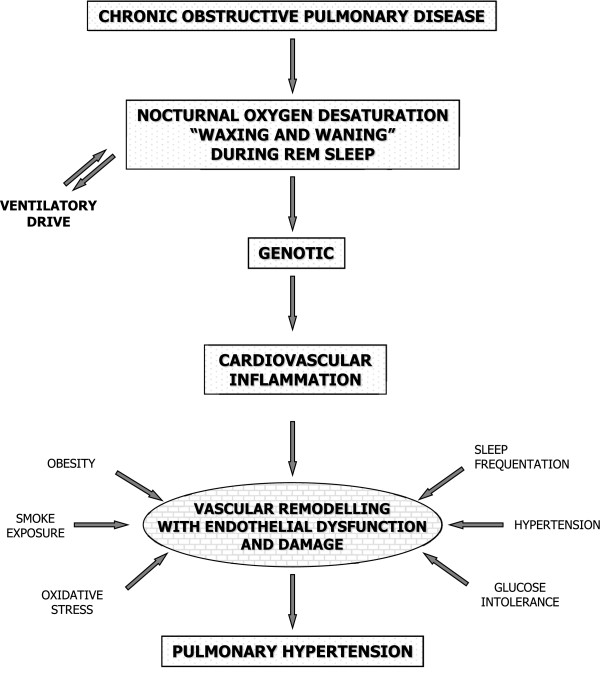
**Biological mechanisms for the development of pulmonary hypertension in COPD patients.** The Diagram shows various processes that drive vasoconstriction, vasodilation, and cell proliferation in the walls of the small pulmonary arteries.

Finally, some studies [40,41] have focused on the role of the alteration of the pulmonary vessels in the development of PH. Hypoxic pulmonary vasoconstriction (HPV) is a physiological self-regulatory response to alveolar hypoxia that distributes pulmonary capillary blood flow to areas of high oxygen availability. This principle is known as the von Euler–Liljestrand mechanism and serves to optimize the flow of blood in the pulmonary capillaries. The alteration of this mechanism may result in hypoxaemia. Under conditions of chronic hypoxia, generalized vasoconstriction of the pulmonary vasculature in concert with nocturnal hypoxia-induced vascular remodelling leads to PH. Reactive oxygen species, redox couplet and adenosine monophosphate-activated kinases are under investigation as mediators of HPV. In addition, experimental data have suggested that several inflammatory proteins play an important role in pulmonary artery physiology and in the regulation of pulmonary artery pressure. However, only a portion of the pathways essential for HPV have been identified and there are still plenty of questions to answer before a complete picture of the oxygen sensing and signal transduction mechanisms of this important physiological response can be elucidated [42] (Figure 3). 

## Conclusion

Patients with COPD frequently experience nocturnal oxygen desaturation episodes, leading to nocturnal hypoxemia. Chronic nocturnal hypoxemia contributes to the development of an adverse sequelae of COPD, including PH, skeletal muscle dysfunction, and cardiovascular disease. Subsequently, the concept of chronic inflammation as one of the key factors involved in pulmonary vascular remodeling has emerged. We propose that data from large clinical trials should be re-analyzed using cluster analysis in order to classify patients according to their clinical characteristics at study entry. However, the clinical application of cluster analysis will depend on developing diagnostic criteria to allow new individuals to be allocated to pre-established clusters. Only time will tell if this new approach is effective in the prediction or diagnosis of COPD-associated diseases and in their prevention or treatment respectively.

## Abbreviations

COPD: Chronic obstructive pulmonary disease; PH: Pulmonary hypertension; HRQoL: Health-related quality of life; SatO_2_: Arterial oxygen saturation; D: Desaturators; ND: Non-desaturators; MPAP: Mean pulmonary arterial pressure; RCH: Right heart catheterization; PAP: Pulmonary arterial pressure; FRC: Functional residual capacity; PaO_2_: Partial pressure of oxygen in arterial blood; RHF: Right heart failure; TRT: Total recording time; PaCO_2_: Partial pressure of CO_2_ in arterial blood; TLC: Total lung capacity; VC: Vital capacity; BMI: Body mass index; HPV: Hypoxic pulmonary vasoconstriction.

## Competing interests

The authors declare that they have no competing interests.

## Authors’ contribution

DT has made a substantial contribution to the conception and design of the study and the analysis and interpretation of the resulting data. GN, MM and FDN were involved in drafting and revising the manuscript critically for important intellectual content and gave final approval of the version to be published.
